# Comparative evaluation of native slow-growing roosters: focus on gut integrity and physiological traits

**DOI:** 10.1186/s12917-025-05084-1

**Published:** 2025-10-17

**Authors:** Eleonora Erika Cappone, Edoardo Fiorilla, Ilario Ferrocino, Marta Gariglio, Valeria Zambotto, Chiara Bianchi, Lara Rastello, Stefano Bagatella, Talal Hassan, Muhammad Adnan Arif, Muhammad Irfan Malik, Ilaria Biasato, Stefania Bergagna, Sara Antoniazzi, Elisabetta Macchi, Isabella Manenti, Kalliopi Rantsiou, Achille Schiavone

**Affiliations:** 1 Department of Veterinary Sciences, University of Turin, Turin, Italy; 2 Department of Agricultural, Forest and Food Science, University of Turin, Turin, Italy; 3 Veterinary Medical Research Institute for Piedmont, Liguria and Aosta Valley, Turin, Italy; 4 Institute of Science of Food Production, National Research Council, Turin, Italy

**Keywords:** Native chicken breeds, Gut health, Corticosterone, Digestibility, Haematological traits, Intestinal histomorphology

## Abstract

Native poultry breeds are fundamental for sustainable farming, especially in agroecological systems emphasizing adaptability, resilience and product quality. However, data on their physiological and intestinal health profiles are limited. This study examines three Italian slow-growing chicken breeds: Bionda Piemontese, Bianca di Saluzzo and Millefiori Piemontese, reared under identical agroecological, free-range conditions. Parameters assessed include productive performance, blood traits, feather corticosterone, intestinal histomorphology and histopathology, nutrient digestibility, and caecal microbiota and volatile fatty acids. All breeds showed similar welfare indicators, with consistent haematological values and feather corticosterone concentrations (*P* > 0.05). While Millefiori Piemontese displayed higher slaughter weight (on average, + 9.10%; *P* = 0.046) and triglycerides (on average, + 21.6%; *P* = 0.021), most intestinal health traits remained comparable among breeds (*P* > 0.05). Analysis of intestinal bacterial composition revealed distinct microbial profiles among the three breeds. Several families and genera varied significantly in relative abundance, indicating breed-associated differences in gut microbiota structure, although overall diversity and fermentation patterns remained similar. These findings provide a useful baseline for future research on the potential role of these breeds in low-input, sustainable farming systems.

**Supplementary Information:**

The online version contains supplementary material available at 10.1186/s12917-025-05084-1.

## Introduction

In recent years, intestinal health in farm animals has gained central importance in assessing welfare, production efficiency, and the final quality of animal products. The intestine, beyond being the main organ for digestion and nutrient absorption, is a complex immunological and microbiological interface where the microbiota plays essential roles in metabolism, immune regulation, and protection against pathogens [1]. In poultry, this delicate balance is influenced by multiple interacting factors such as diet, microbiome composition, and environmental conditions, which together shape overall health and productivity. Alterations in intestinal structure or microbial communities can compromise health, increasing susceptibility to infections, environmental stress, and nutritional inefficiencies [2, 3]. Therefore, maintaining a balanced microbiome is crucial to prevent pathogenic infections, promote efficient nutrient utilisation, and sustain immune function. To this end, the study of volatile fatty acids and the inflammatory status of critical organs such as the intestinal mucosa, liver, and spleen provides essential insights into gut functionality and the systemic implications of intestinal health [4, 5]. Achieving these goals requires a holistic, systems-based approach that integrates dietary strategies, microbiome management, and host immune responses, recognising the intricate nature of the gut ecosystem and the need for tailored interventions to optimise intestinal health outcomes [6].

Thanks to the significant progress made in recent years in the genetic characterization of local Italian poultry breeds, such as Bionda Piemontese (BP), Bianca di Saluzzo (BS) and Millefiori Piemontese (MP) (Fig. S1), we now have a solid genetic base on which to build new research paths [7–10]. This information heritage opens promising opportunities to also delve into the physiological, immunological and intestinal health aspects of these breeds, to fully understand their potential. Integrating genetic knowledge with functional data allows for targeted studies in agroecological contexts, in which the ability of animals to adapt to dynamic and less standardized environmental conditions represents a strategic value [11]. Building a clear and multidimensional baseline in controlled conditions is therefore a key step to enhance the adaptability and resilience of these breeds within sustainable production systems.

In parallel, interest in more sustainable and welfare-friendly farming systems has led to a renewed appreciation for native poultry breeds, particularly slow-growing ones, historically associated with rural and local contexts, making them well-suited for agroecological systems [12, 13]. The protection and enhancement of native breeds do not only respond to a cultural or genetic biodiversity need but also takes on a strategic value in the zootechnical and environmental fields. In fact, native breeds represent an important resource for the development of sustainable breeding models, capable of enhancing local resources, reducing dependence on external inputs and ensuring greater resilience to environmental stress. Furthermore, their conservation is in line with European and international policies that promote the protection of animal genetic resources as an integral part of food security and agroecological sustainability [12, 14, 15].

These breeds, often neglected by commercial selection programs, represent a genetic heritage of great value, not only for their adaptability to less intensive environmental conditions, but also for the qualitative characteristics of their production, in particular meat [7, 16]. However, while the scientific literature is rich in studies focused on broiler lines selected for high performance in controlled environments, knowledge about the physiology, intestinal health and microbiota of native breeds is still fragmented [4]. In Italy, three of the main slow-growing native chicken breeds that deserve particular attention are Bionda Piemontese, Bianca di Saluzzo and Millefiori Piemontese. These breeds, originating from the Piedmont region (north-west Italy), are the result of centuries-old processes of natural and traditional selection, and are characterized by their good adaptability to outdoor farming, rusticity and high organoleptic quality of meat [7]. The Bionda Piemontese is recognizable by its golden and uniform plumage, and is traditionally bred for mixed production, both of meat and eggs. The Bianca di Saluzzo, characterized by a pure white plumage, is known for its good performance in terms of growth and quality of the breast. The Millefiori Piemontese, with its variegated plumage, is instead appreciated for its extreme rusticity and foraging ability in extensive systems [17]. Currently all three are the subject of genetic conservation and enhancement programs, as they are considered at risk of genetic erosion due to the progressive replacement with industrial hybrid breeds [18, 19].

In this scenario, the present study aims to comparatively analyse these three slow-growing native breeds, to explore their physiological and intestinal characteristics. The investigation includes the evaluation of productive performance, blood chemistry and metabolic parameters, corticosterone levels in feathers as an indicator of chronic stress, intestinal morphology, nutrient digestibility, and the composition of the caecal microbiota [20–23]. This multidimensional approach allows to obtain a holistic view of the health status of the animals, highlighting any breed peculiarities in terms of metabolism, adaptation and interaction with the intestinal environment. The results obtained may contribute to the broader understanding of how native breeds perform under different rearing conditions, offering useful insights for the development of more targeted nutritional and management strategies. In the context of a rapidly evolving agricultural landscape, recognising and exploring the specific traits of local breeds could represent a valuable step toward integrating productivity, animal welfare, and biodiversity conservation within alternative, high-value supply chains.

## Materials and methods

### Birds and husbandry

The experimental trial was conducted at the poultry facility of the Avian Conservation Centre for the Valorisation of Local Genetic Resources (CoVaGEN) at the University of Turin, Italy. The study protocol (Prot. No. 0544534 of 23/09/2024) was approved by the University of Turin’s Bioethical Committee. Birds were given *ad libitum* access to water and a commercial corn–soybean meal–based feed (Fratelli Borello S.p.A., Bra, Cuneo, Italy), administered in three phases: from day 1 to 28 (AMEn: 12.5 MJ/kg; CP: 22.0%, as fed; Ca: 0.97%; available P: 0.49%; Lys: 1.42%; Met: 0.73%; Met + Cys: 1.09%), from day 29 to 84 (AMEn: 12.8 MJ/kg; CP: 20.5%, as fed; Ca: 0.85%; available P: 0.44%; Lys: 1.33%; Met: 0.75%; Met + Cys: 1.10%), and from day 85 to 182 (AMEn: 13.3 MJ/kg; CP: 18.0%, as fed; Ca: 0.75%; available P: 0.38%; Lys: 1.23%; Met: 0.71%; Met + Cys: 1.02%).

At the age of 29 d, a total of 160 male birds were housed in pens according to breed, with ten birds per pen and six replicates for BP and BS and four replicates for MP. Each pen measured 6 m × 1.5 m × 10 m (l × w × h) and rice hulls was used as litter. The facility operated under natural environmental conditions, and the trial was carried out from May to November 2023. During this period, the average monthly temperatures ranged from approximately 20 °C in May, 25 °C in June, 28 °C in July, 27 °C in August, 22 °C in September, 17 °C in October, to 12 °C in November. Relative humidity varied between 60 and 75%, while the natural photoperiod ranged from about 15 h of natural daylight in June to 9.5 h in November. Vaccination protocols followed standard poultry health programs, including protection against Marek’s disease, Newcastle disease, and infectious bronchitis. Veterinary care was provided throughout the trial, with routine monitoring to ensure flock health and welfare. At 182 days of age, 15 males per breed were selected for slaughter based on average weights. The selected birds were weighed prior to slaughtering to determine their slaughter weight (SW), then electrically stunned and exsanguinated in compliance with EU regulations (Council Regulation (EC) No 1099/2009); blood was collected in EDTA tubes during the exsanguination phase. Carcasses were plucked, eviscerated, and weighed to record the ready-to-cook carcass (RTCC) weight. Additionally, the spleen and liver were weighed post-evisceration for the calculation of their relative weights (% SW). All measurements were taken using electronic scales (KERN PLE-N v. 2.2, Balingen, Germany).

### Corticosterone metabolites in feathers and haematological traits

For the quantification of corticosterone in feathers, 45 (15/breed) samples were collected from the interscapular region at the time of experimental slaughter following the procedures by Temple et al. [24]. From each individual, five to eight fully grown feathers were collected, selecting only those with a minimum length of 30 cm and a mass of at least 10 mg to ensure adequate hormone extraction. Samples were stored at room temperature until analysis. Corticosterone extraction was performed following protocols of [25–27] with slight changes. The calamus was removed, keeping a feather’s section of 70–80 mm, and then they were cut with scissors into very small pieces (< 5 mm^2^). 3 mL of 100% methanol was added to the samples. The samples with methanol were then incubated at 50 °C for 24 h. Feather samples and methanol was separated using a 0.22 μm syringe filter. To avoid loss of corticosterone extracted, feather’s pieces were then vortexed for 30 s with 1 mL extra of methanol that was subsequently filtered and added to formerly filtered methanol from the sample. The methanol was then evaporated under a fume hood overnight and samples were stocked at −20 °C until analyses. For the quantification of corticosterone, 200 µL of assay diluent buffer provided by the ELISA kit (K014-H, Arbor Assay, Ann Arbor, MI, USA) were added to the dried hormone extracted from the feather samples and analyses were performed following manufacturer’s instructions. The kit’s values of cross-reactivity, as reported by the manufacturer, were: 100% for corticosterone, 12.3% for deoxycorticosterone, 0.62% for aldosterone, 0.38% for cortisol, and 0.24% for progesterone.

### Histomorphological and histopathological investigations

After slaughtering, 45 (15/breed) samples were submitted to anatomopathological investigations. For histological evaluation, a 5-cm segment of the small intestine was collected in a standardized way: from the duodenum at the duodenal loop, from the jejunum immediately before Meckel’s diverticulum (representing the mid-jejunum), and from the ileum immediately before the ileocecal junction. These anatomical landmarks were chosen to ensure that comparable regions were sampled across all animals and treatments. After sample collection the excised tissues were flushed with 0.9% saline to remove all the content and 10% formalin fixed for histomorphometric analysis. Tissue sections were processed and dehydrated through a graded series of ethanol (70%, 80%, 95%, and 100%). After that clearance of the tissue were carried out in isoparaffin, they were embedded in paraffin wax. The tissue sectioning of the wax embedded tissue was carried out at 5 μm thickness with microtome. The tissue section mounted on glass slides were stained with Haematoxylin & Eosin (HE). One slide per intestinal segment was examined by light microscopy and each slide was captured with a Nikon DS-Fi1 digital camera (Nikon Corporation, Tokyo, Japan) coupled to a Zeiss Axiophot microscope (Carl Zeiss, Oberkochen, Germania) using a 2.5× objective lens. The NIS-Elements F software was used for image capturing and morphometric analysis was performed by Image^®^-Pro Plus software (6.0 version, Media Cybernetics, Maryland, USA) [28]. Slides were evaluated for morphometric measurements of VH (from the tip of the villus to the crypt), CD (from the base of the villus to the submucosa) and the Vh/Cd ratio [29]. For morphometric measurements 10 well-oriented and intact villi were selected, for the crypt depth, 10 crypts were chosen from duodenum, jejunum and ileum [28]. The mucosal, submucosal and muscular thickness were also measured on 3 standardized points of the gut layers per each captured field. Similarly, for histological evaluation, standardized sampling points were used for liver and spleen. A portion of the left lobe was collected from the liver, while a transverse section was obtained from the middle part of the spleen. The following histopathological alterations were evaluated: gut inflammation, inflammation, white pulp hyperplasia and depletion in the spleen, and inflammation and hepatocyte degeneration in the liver. All the observed histopathological alterations were evaluated using a semiquantitative scoring system as follows: absent (score = 0), mild (score = 1), moderate (score = 2), and severe (score = 3). Inflammatory infiltrates were also assessed considering type and pattern for each gut segment as follow: absent (score = 0), mononuclear (score = 1), mixed with neutrophils (score = 2), and mixed with eosinophils (score = 3), and absent (score = 0), focal (score = 1), multifocal (score = 2), disseminated (score = 3), and diffuse (score 4) for type and pattern respectively.

### Nutrient digestibility

Titanium dioxide (TiO₂) was incorporated into the feed as an inert marker at a concentration of 0.5% to facilitate the assessment of ileal nutrient digestibility. At the conclusion of the trial, after slaughtering the ileum content samples were then pooled by pen (5 replicates) and immediately frozen at −20 °C to preserve their integrity for subsequent laboratory analysis. Before analysis, the samples were freeze dried and finely ground using a laboratory mill to pass through a 0.5 mm sieve.

TiO₂ concentration was determined through acid digestion, where samples undergo oxidation using concentrated sulfuric acid (H₂SO₄) and hydrogen peroxide (H₂O₂) [30]. The process involves heating at 250 °C to break down organic matter, followed by dilution and colour development for spectrophotometric analysis. The resulting solution’s absorbance is measured at 410 nm using a UV-Vis spectrophotometer, allowing quantification of TiO₂ for digestibility calculations. A calibration curve is established using TiO₂ standard solutions of known concentrations to ensure accurate quantification. The TiO₂ concentration in feed and digesta is determined based on the calibration curve. To ensure accuracy and reliability, all sample analyses are performed in triplicate. Reagent blanks and TiO₂ standard solutions are included in the analysis to validate the spectrophotometric measurements. The apparent total tract digestibility coefficient (ATTDC) of dietary nutrients was determined using the following calculation:

$$\:Digestibility\:=\:\left[\frac{\left(\%\:{X}_{diet}\:/\:\%\:{TiO2}_{\:diet}\right)-\:\left(\%\:{X}_{excreta}/\:\%\:{TiO2}_{excreta}\right)}{\left(\%\:{X}_{diet}\:/\:\%\:{TiO2}_{\:diet}\right)}\right]$$

where X represents crude protein (CP), or ether extract (EE).

CP content in feed and digesta was determined by measuring total nitrogen using the Kjeldahl method, following the AOAC Official Method 2001.11, and applying a nitrogen-to-protein conversion factor of 6.25. For EE, the analysis was carried out according to the Soxhlet extraction method, using petroleum ether as solvent, in accordance with AOAC Official Method 920.39. All analyses were performed in triplicate to ensure precision and reproducibility. The results were expressed as percentages on a dry matter basis, after drying the samples at 105 °C to constant weight.

### Microbiota

Caecum content samples were collected after slaughtering using sterile equipment to prevent contamination. These samples were immediately frozen at −80 °C for storage until further analysis. Total DNA was extracted from the caecal samples and analysed using a metataxonomic approach to examine potential variations in microbiota composition. The 16 S rRNA gene, specifically targeting the V3-V4 regions, was amplified, purified, tagged, and pooled according to Illumina’s guidelines [31]. The sequencing was performed on the Illumina MiSeq platform using V2 chemistry, generating 250-bp paired-end reads. The resulting raw fastq files were processed using QIIME 2 software [32]. Following the methodology outlined by Callahan et al. [33], primer sequences were removed using the Cutadapt tool, and the DADA2 algorithm was applied for denoising reads through the q2-dada2 plugin within the QIIME 2 environment. Taxonomic classification was conducted using the QIIME 2 feature-classifier against the SILVA database. To enhance the reliability of sequence reads, amplicon sequence variants (ASVs) with a read count of fewer than five in at least two samples were excluded.

### Intestinal volatile fatty acids

The same procedure used to collect caecal content for microbiota analysis was also applied to obtain samples for volatile fatty acid (VFA). Specifically, 300 mg of caecal content was suspended in 600 µL of 0.1 N H₂SO₄ and vortexed thoroughly. The mixture was then centrifuged at 15,000 × g for 10 min at 4 °C, and the resulting supernatant was transferred into 2 mL clear glass vials (Sigma-Aldrich) [5]. VFA concentrations were measured using High-Performance Liquid Chromatography (HPLC) with a Dionex UVD 340U UV/VIS detector (Thermo Fisher), equipped with a 300 × 7.8 mm Aminex HPX-87 H column (Bio-Rad) and a corresponding guard column. A 30 µL sample was injected and separated isocratically using 0.005 N H₂SO₄ as the mobile phase, with a flow rate of 0.6 mL/min at 41 °C. Detection was performed at 210 nm, and quantification was based on an external standard curve. Standards were prepared in 0.1 N H₂SO₄ and covered the following concentration ranges: 4.95–148.5 mg/100 mL for succinic acid, 9–270 mg/100 mL for lactic acid, 10.5–314.4.5.4 mg/100 mL for acetic acid, 9.85–285.5 mg/100 mL for propionic acid, 9.4–282.1 mg/100 mL for butyric acid, 9.5–285.1 mg/100 mL for isobutyric acid, 9.1–273.4 mg/100 mL for isovaleric acid, 9.1–273.2 mg/100 mL for valeric acid, and 4.95–148.5 mg/100 mL for citric acid.

### Data analysis

Corticosterone metabolites in feathers, haematological traits, and nutrient digestibility were analysed using a one-way analysis of variance (ANOVA), with breed as the fixed factor. When significant effects were detected (*P* ≤ 0.05), Tukey’s Honest Significant Difference (HSD) test was applied post hoc to identify pairwise differences between group means. Data are presented as mean values with their standard errors (SEM).

Gut morphometric measurements were analysed using a generalized linear mixed model (GLMM), in which breed, and intestinal segment were included as fixed factors, and pen was included as a random factor to account for repeated measures within experimental units. When significant effects or interactions were observed, Tukey’s HSD test was applied for multiple comparisons (α = 0.05).

For microbiota data, different statistical approaches were applied according to the type of analysis. Alpha-diversity indices (Shannon, Simpson, Chao1) were compared among breeds using one-way ANOVA. Beta-diversity was assessed through permutational multivariate analysis of variance (PERMANOVA) based on Bray-Curtis dissimilarity matrices and visualized by Principal Component Analysis (PCA) plots. To evaluate differences in the relative abundance of bacterial taxa among breeds, the non-parametric Kruskal-Wallis test was used, followed, when appropriate, by Dunn’s post hoc test with Bonferroni-adjusted P-values to control for Type I error.

All statistical analyses were performed using R software (version 4.4.0), with the packages vegan (for multivariate analyses) and FSA (for non-parametric multiple comparisons).

## Results

### Slaughtering performance, corticosterone metabolites in feathers and haematological traits

The analysis of productive performance and haematological parameters in the BP, BS and MP breeds showed significant differences in some indicators (Table 1).

**Table 1:** Comparison of productive performance and haematological parameters in roosters of Bionda Piemontese (BP), Bianca Di Saluzzo (BS), and millefiori Piemontese (MP) chicken breeds (least square means value; standard error of mean, SEM)

	BP	BS	MP	SEM	*P*-value
Productive performance					
Live weight, 0 d (g)	40.8	40.9	36.3	2.782	0.061
Live weight, 31 d (g)	421^a^	376^b^	332^b^	12.78	< 0.001
Slaughter weight, 182 d (g)	3034^b^	2912^b^	3242^a^	111.7	0.046
Ready to cook carcass (%)	66.9	65.0	65.6	1.383	0.374
Haematological traits					
Cholesterol (mg/dl)	125	128	129	4.059	0.903
Triglycerides (mg/dl)	56.5^b^	51.2^b^	65.6^a^	3.785	0.021
Creatinine (mg/dl)	0.211	0.185	0.201	0.06	0.335
Uric Acid (mg/dl)	5.42	3.94	5.24	0.688	0.152
Total Proteins (mg/dl)	4.55	5.21	5.35	0.421	0.183
Phosphorus (mg/dl)	5.21	5.24	5.46	0.262	0.741
Calcium (mg/dl)	11.1	11.7	10.7	0.28	0.374
Iron (mg/dl)	211	196	190	10.91	0.382
Magnesium (mg/dl)	3.49	3.68	3.46	0.153	0.571
Gamma-glutamyl transferase (U/L)	28.8	30.1	30.5	1.589	0.768
Aspartate aminotransferase (U/L)	180	183	191	5.352	0.448
Alanine aminotransferase (U/L)	3.15	2.86	2.71	0.516	0.718

In terms of productive performance, live weight at 31 days was significantly higher in BP roosters (421 g) compared to BS (376 g) and MP (332 g) (*P* < 0.001). Conversely, slaughter weight (SW) was significantly greater in MP (3242 g) than in BP (3034 g) and BS (2912 g) (*P* = 0.046). No significant differences were observed among breeds for the RTCC weight (*P* = 0.374).

**Fig. 1:**
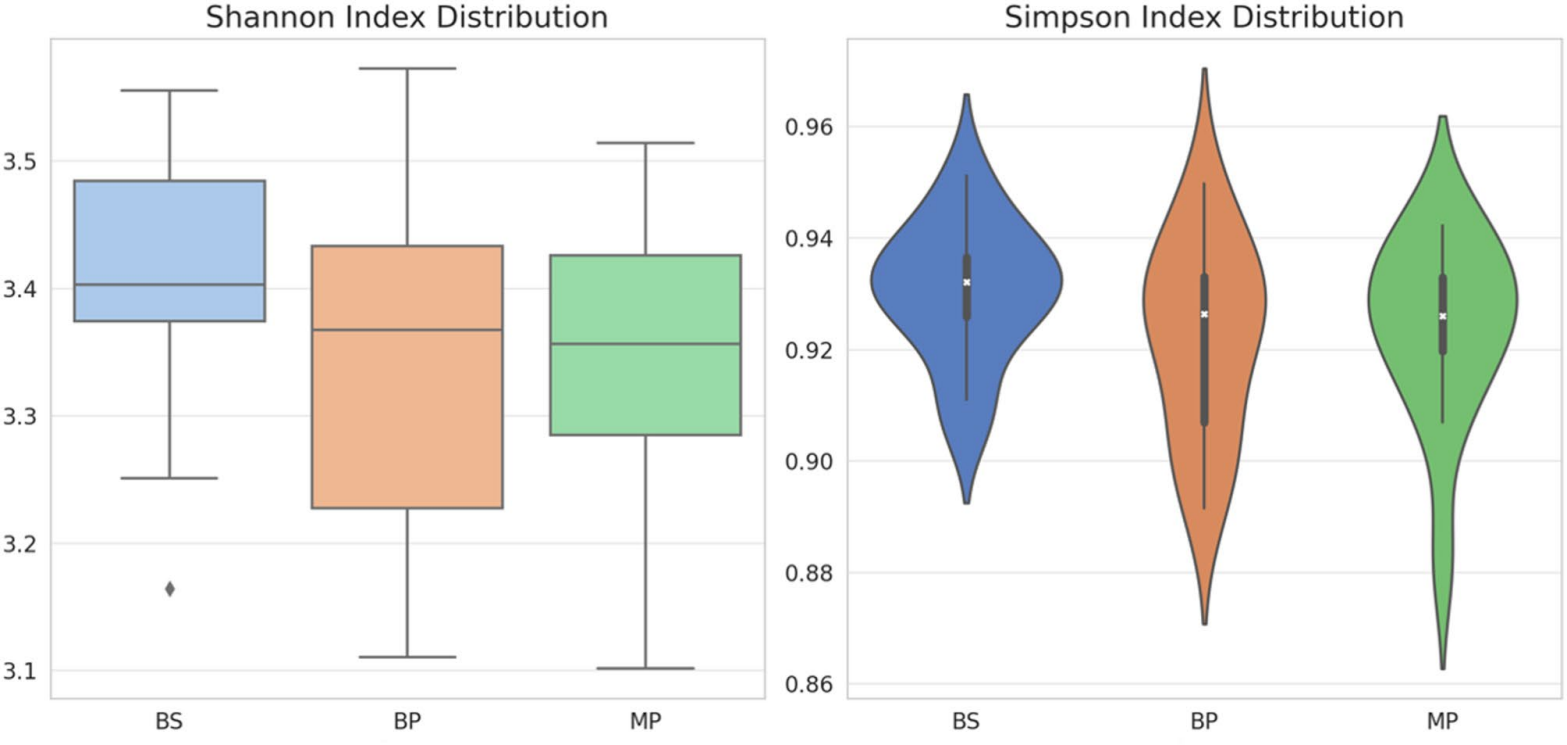
Corticosterone metabolite values (CMF) in the feathers of Bionda Piemontese (BP), Bianca di Saluzzo (BS), and Millefiori Piemontese (MF) chicken breeds

Haematological analyses revealed no significant differences among breeds except for triglyceride, since MP roosters exhibited significantly higher values (65.6 mg/dL) than BP (56.5 mg/dL) and BS (51.2 mg/dL) (*P* = 0.021). Similarly, no statistically significant differences were found in corticosterone metabolite levels in feathers (CMF) among the three breeds (*P* = 0.227), with average values of 60.37 ng/g for BP, 63.22 ng/g for BS, and 55.71 ng/g for MP (Fig. 1). Detailed data are available in Table S1.

### Histomorphological investigations, blood immune markers and nutrient digestibility

The results of the morphometric measurements and inflammation scoring of duodenum, jejunum and ileum are reported in Table 2. No significant interactions were found for any of the evaluated morphological parameters, including villus height, villus width, crypt depth, villus height to crypt depth ratio (VH: CD), mucosal thickness, submucosal thickness, and muscularis thickness (*P* > 0.05 for all interactions).

**Table 2:** Morphometric measurements (mm) and inflammation scoring of duodenum, jejunum and ileum in roosters of Bionda Piemontese (BP), Bianca Di Saluzzo (BS), and millefiori Piemontese (MP) chicken breeds (least square means value; standard error of mean, SEM)

	BS	BP	MP	SEM	*P*-value
Duodenum					
Villus height	0.57	0.61	0.59	0.012	0.514
Villus width	0.08	0.08	0.08	0.024	0.572
Crypt depth	0.11	0.10	0.12	0.020	0.936
Villus height/crypt depth	5.10	4.91	5.12	0.173	0.819
Mucosal thickness	0.78	0.79	0.78	0.018	0.257
Sub-mucosal thickness	0.05	0.06	0.06	0.008	0.782
Muscularis thickness	0.11	0.12	0.12	0.006	0.811
Jejunum					
Villus height	0.49	0.48	0.49	0.022	0.356
Villus width	0.07	0.07	0.06	0.017	0.526
Crypt depth	0.10	0.09	0.09	0.003	0.385
Villus height/crypt depth	4.10	4.20	4.20	0.351	0.677
Mucosal thickness	0.51	0.50	0.50	0.024	0.464
Sub-mucosal thickness	0.04	0.04	0.04	0.022	0.522
Muscularis thickness	0.12	0.12	0.12	0.009	0.585
Ileum					
Villus height	0.53	0.55	0.54	0.019	0.211
Villus width	0.07	0.07	0.07	0.002	0.329
Crypt depth	0.12	0.12	0.12	0.011	0.618
Villus height/crypt depth	4.40	4.60	4.40	0.251	0.417
Mucosal thickness	0.77	0.75	0.76	0.014	0.401
Sub-mucosal thickness	0.06	0.06	0.06	0.008	0.523
Muscularis thickness	0.12	0.12	0.12	0.007	0.139

Similarly, there were no differences regarding inflammation pattern nor severity. Histopathological scoring of liver and spleen are reported in Table 3. Liver inflammation pattern, type, severity and degeneration were not influenced (*P* > 0.05) by breed type. The inflammation score for spleen was not present in any samples in all breeds. The spleen hyperplasia and depletion were also non-significant (*P* > 0.05) in all chicken breeds.

**Table 3:** Histopathological scoring of small intestine, liver and spleen in roosters of Bionda Piemontese (BP), Bianca Di Saluzzo (BS), and millefiori Piemontese (MP) chicken breeds (least square means value; standard error of mean, SEM)

	BS	BP	MP	SEM	*P*-value	
Small intestinal inflammation score						
Inflammation Pattern	3.33	3.03	3.3	0.255	0.332	
Inflammation severity	0.96	0.87	0.94	0.13	0.535	
Liver						
Inflammation pattern	2	2	2	--	--	
Inflammation type	1.71	1.5	1.46	0.132	0.361	
Inflammation severity	1.02	1.07	1.00	0.117	0.341	
Degeneration	0.643	0.857	0.933	0.176	0.350	
Spleen						
Inflammation pattern	0	0	0	--	--	
Inflammation type	0	0	0	--	--	
Inflammation severity	0	0	0	--	--	
Hyperplasia	1.3	1.5	1.5	0.119	0.492	
Depletion	0.83	0.9	0.9	0.141	0.896	

Finally, protein digestibility was 50.2% in BP, 54.6% in BS, and 52.5% in MP (*P* = 0.641). Ether extract digestibility was higher in all breeds, with values of 74.2% in BP, 80.2% in BS, and 78.4% in MP (*P* = 0.209).

### Microbiota

**Fig. 2:**
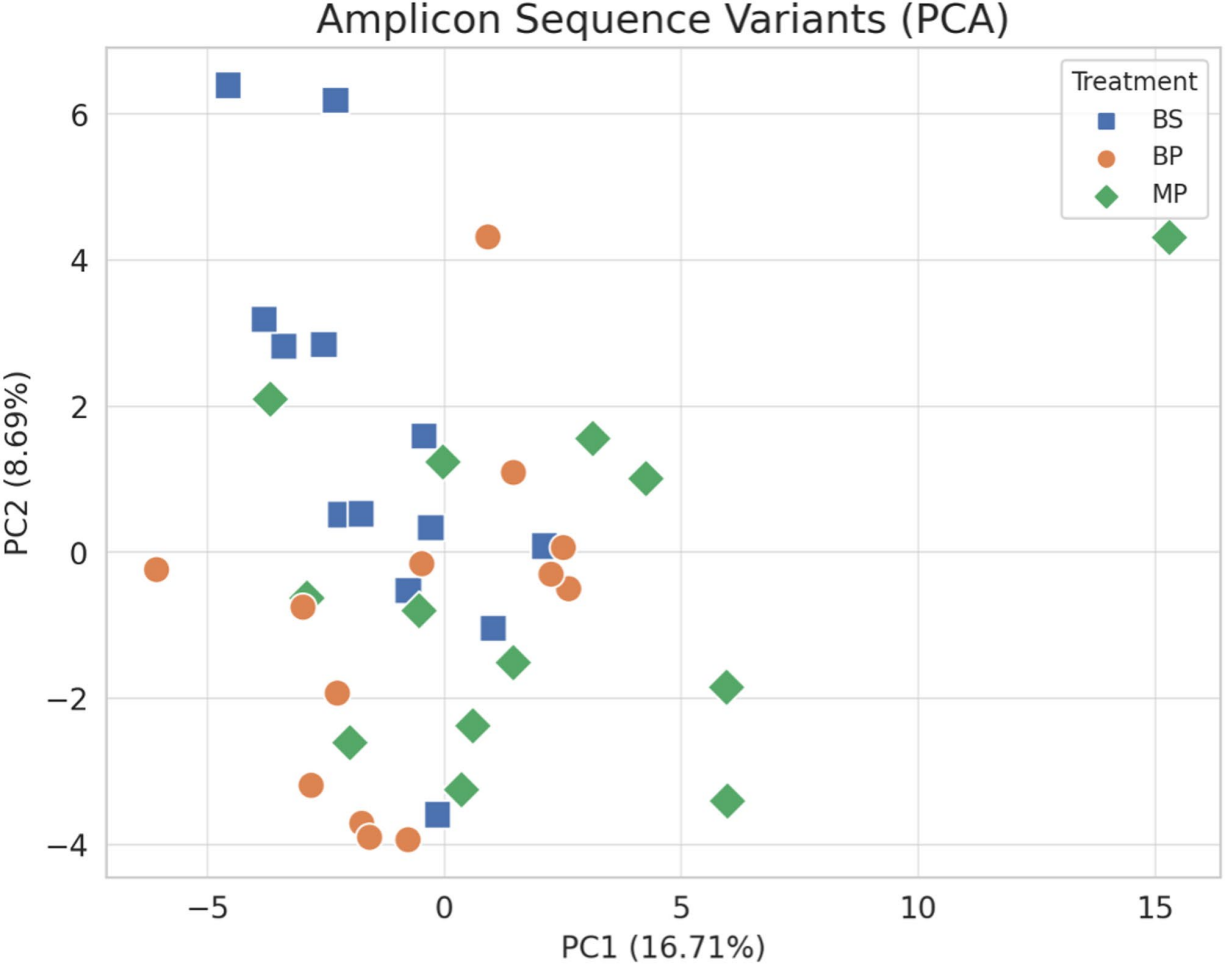
Microbiota diversity index in the gut of roosters of Bionda Piemontese (BP), Bianca di Saluzzo (BS), and Millefiori Piemontese (MP) chicken breeds

The analysis of alpha diversity of the intestinal microbiota in roosters of the BP, BS, and MP breeds, measured by the Simpson and Shannon indices, did not reveal significant differences among groups (*P* > 0.05; Fig. 2).

**Fig. 3:**
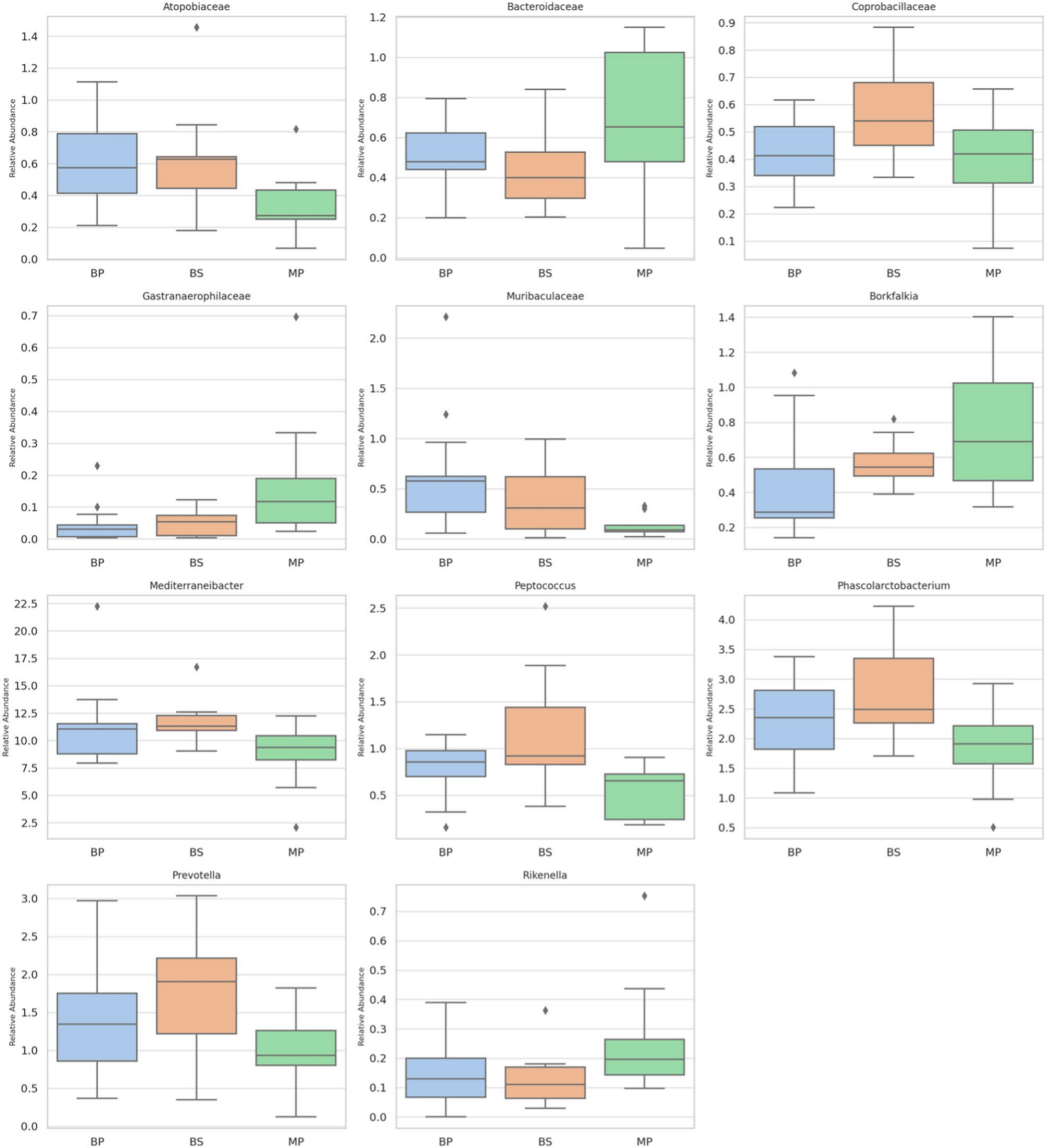
Principal component analysis (PCA) of amplicon sequence variant (ASV) in the gut of roosters of Bionda Piemontese (BP), Bianca di Saluzzo (BS), and Millefiori Piemontese (MP) chicken breeds

Beta-diversity analysis, based on Bray–Curtis dissimilarity, was statistically evaluated using PERMANOVA (999 permutations), which indicated no significant differences among breeds (F = 24.06; R² = 0.572; *P* = 0.061), although a trend toward separation was observed. Principal Coordinate Analysis (PCoA) was additionally used for visualization purposes, and the ordination plot (Fig. 3) showed a substantial overlap of samples from the three breeds, without clear group separation in the composition of the intestinal microbiota.

**Fig. 4:**
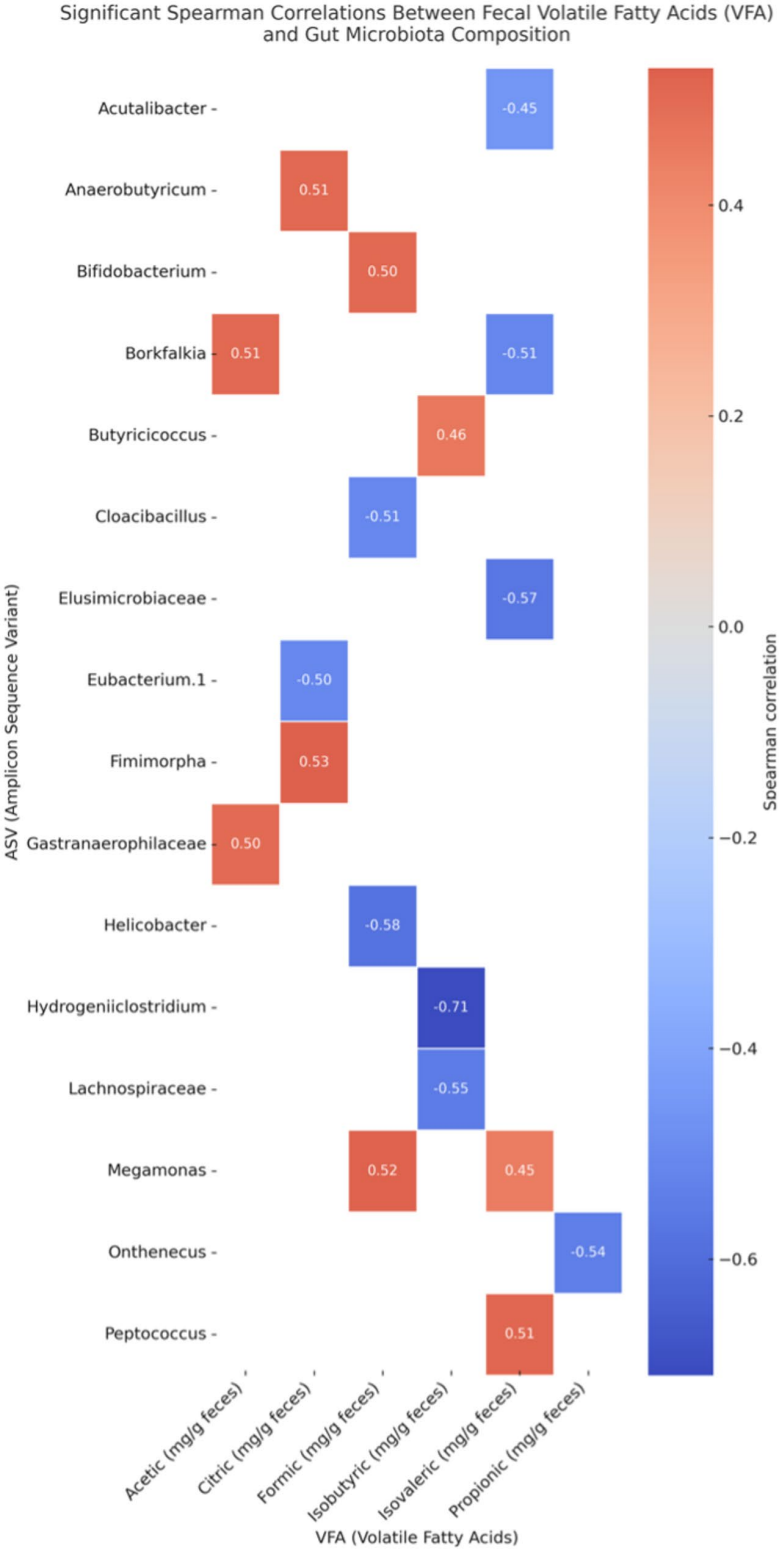
Different bacteria in the gut of roosters Bionda Piemontese (BP), Bianca di Saluzzo (BS), and Millefiori Piemontese (MP) chicken breeds

The analysis of intestinal bacterial composition highlights differences among the breeds, indicating distinct microbial profiles (Fig. 4). Notably, *Atopobiaceae* was found to be less abundant in MP compared to BP and BS (*P* = 0.018). Conversely, *Bacteroidaceae* was more prevalent in MP than in the other two breeds (*P* = 0.026). Further distinctions emerged in *Coprobacillaceae*, which exhibited a higher presence in BS than in BP and MP (*P* = 0.018), and *Gastranaerophilaceae*, which was more abundant in MP (*P* = 0.018). Similarly, *Muribaculaceae* was more prominent in BP, whereas *Borkfalki*a showed a higher concentration in MP (*P* = 0.010) Interestingly, *Mediterraneibacter* was found in lower abundance in MP compared to BP and BS (*p* = 0.031), while *Peptococcus* and *Prevotella* were both more prevalent in BS (*P* = 0.003 and *p* = 0.033, respectively). Additionally, *Rikenella* appeared in higher quantities in MP (*P* = 0.039). Although was detected at a higher level in BS compared to BP and MP, the difference did not reach statistical significance (*P* = 0.065).

### Intestinal volatile fatty acids

Mean concentrations for all VFAs are summarized in Table 4. Overall, the concentrations of volatile fatty acids (VFAs) were comparable among the three breeds, with no major differences observed for most compounds. However, isobutyric acid levels were significantly higher in BP than MP and BS (*P* = 0.043), and n-valeric acid was higher in BS compared to MP and BP (*P* = 0.039).

**Table 4:** The volatile fatty acids (mg/g excreta) in the gut of Bionda Piemontese (BP), Bianca Di Saluzzo (BS), and millefiori Piemontese (MP) chicken breeds (least square means value; standard error of mean, SEM)

	BS	BP	MP	SEM	*P*-value
Citric	0.055	0.041	0.048	0.0072	0.536
Succinic	0.270	0.203	0.250	0.0488	0.533
Formic	0.066	0.105	0.076	0.0203	0.351
Acetic	4.275	3.796	5.378	0.4420	0.072
Propionic	0.059	0.099	0.048	0.0474	0.449
Isobutyric	0.213^b^	0.355^a^	0.306^a^	0.0407	0.043
Butyric	0.115	0.184	0.175	0.0630	0.569
Isovaleric	0.058	0.062	0.034	0.0081	0.365
N-valeric	0.509^a^	0.268^b^	0.488^a^	0.0331	0.039

**Fig. 5:**
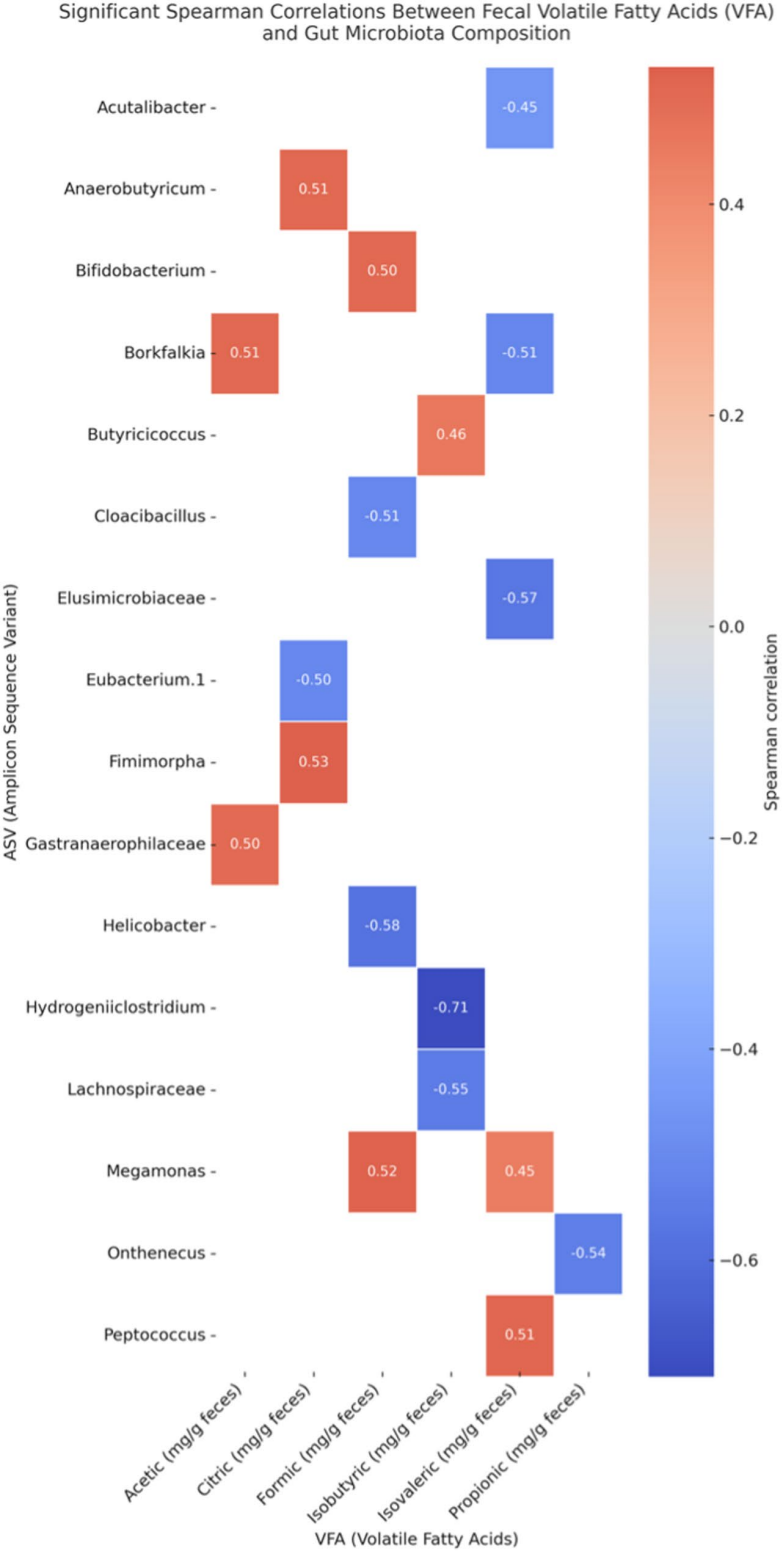
Spearman correlations between Faecal Volatile Fatty Acids (VFA) and Gut Microbiota (ASV) composition in the gut of roosters Bionda Piemontese (BP), Bianca di Saluzzo (BS), and Millefiori Piemontese (MP) chicken breeds

All significant correlation values are displayed in Fig. 5. Significant positive correlations were observed between citric acid and *Fimimorpha* (*r* = 0.53, *p* = 0.013), formic acid and *Megamonas* (*r* = 0.52, *p* = 0.015), isovaleric acid and *Peptococcus* (*r* = 0.51, *p* = 0.018), and acetic acid and *Borkfalkia* (*r* = 0.51, *p* = 0.018).

## Discussion

The results highlight some interesting similarities among three native slow-growing poultry breeds, both in terms of productive performance and regarding the haemato-biochemical profiles and the levels of corticosterone detected in the feathers. From the point of view of growth performance, the finding that MP reaches a significantly higher SW than BS and BP despite starting from a lower initial weight indicates a slower growth in the initial stages but the achievement of higher final weights. This difference is typical of slow-growing native breeds, with variable growth rates depending on the life stage among the different breeds [7–9, 19]. Regarding the haemato-biochemical parameters, an overall situation of homogeneity is found among the groups, except for triglycerides, which were higher in MP. This finding suggests subtle differences in lipid metabolism in MP roosters, potentially linked to the greater energy demand required to achieve their higher final body weight. The elevated triglyceride levels observed in our study could reflect an increased capacity for lipid synthesis or mobilization to support prolonged growth [34] or a more efficient fat storage or utilization strategy, possibly associated with breed-specific metabolic traits [35]. However, these differences appeared limited, as no significant variations were found in cholesterol, creatinine, uric acid, total protein, minerals or liver enzymes, indicating a comparable physiological and metabolic status across breeds [36].

The absence of significant differences in CMF levels between breeds suggests a similar physiological response to the free-range rearing conditions. This uniformity may reflect a comparable capacity across genotypes to maintain welfare under such environmental settings. Given that feathers incorporate circulating corticosterone during growth, CMF provides an integrated measure of cumulative glucocorticoid exposure over the feather development period [37].

Moreover, the results from morphometric analyses, nutrient digestibility, and VFA profiles provide useful baseline information on the evaluated parameters, while the genetic background of the breeds appears to have a limited influence on most of them. This consistent absence of significant breed-related differences across structural, functional, and fermentative parameters supports the hypothesis of a shared physiological framework governing intestinal development, digestion, and microbial activity. Morphometric changes are key indicators of intestinal health and inflammation. Stefanetti et al. [21] found that farming systems affect intestinal morphology and immunity, with native breeds adapting better to free-range and low-input diets than commercial hybrids like Ross 308. In our study, villus height was not significantly affected by breed (*P* >0.05) while some studies explore the role of breed in villus height and absorption, evidence shows that, despite genetic selection affecting morphology and gene expression, breed does not significantly influence villus height. For instance, comparisons between White Plymouth Rocks and commercial broilers showed morphological and gene expression differences, but not in villus height [1]. The absence of significant breed-related differences in these parameters indicates that intestinal development is predominantly governed by conserved regulatory mechanisms rather than breed-specific genetic determinants [38]. Analysis of intestinal inflammation showed that the pattern, type, and severity of inflammation were not significantly influenced by breed or intestinal region, and the observed values were within the physiological ranges previously reported in similar breeds [4]. Moreover, the complete absence of splenic inflammation in all groups further supports the overall low inflammatory status. The analysis of ileal digestibility of CP and EE confirms previous findings, indicating that breed is not a major influencing factor. Across all genotypes, lipid digestibility was consistently higher than that of protein. This aligns with existing literature, which attributes the more efficient absorption of lipids to their distinct digestion and uptake mechanisms in the small intestine [39]. Despite its relevance, nutrient digestibility has been scarcely investigated in native, slow-growing breeds. Nevertheless, comparisons can be drawn with poultry reared over longer production cycles, such as laying hens and roosters. Huang et al. [40] reported protein digestibility values of 79% in 55-week-old Isa Brown laying hens and 71% in 44-week-old roosters, both of which were higher than those observed in the native breeds evaluated in the present study. Similarly, Mtei et al. [41] found a protein digestibility of 67% in 59-week-old Hy-Line Brown laying hens, which also exceeded the values obtained for the three Piedmontese breeds. In contrast, lipid digestibility in the same Hy-Line Brown hens was 57%, which was lower than that recorded in the present study.

Furthermore, analysis of gut microbiota composition revealed a similarity in the richness and homogeneity of the intestinal microbial community among the three breeds, as indicated by alpha diversity indices and PCA. However, differences in the abundance of specific bacterial taxa suggest potential host-microbiota interactions. For instance, the lower abundance of *Atopobiaceae* in MP compared to BP and BS may reflect variations in intestinal fermentation or carbohydrate metabolism. Similarly, the higher abundance of *Bacteroidaceae* in MP chickens may reflect a greater capacity for fibre degradation relative to other breeds, due to the known role of this family in breaking down complex carbohydrates and producing VFAs, which are essential for plant fibre fermentation [22]. This is further supported by their involvement in the degradation of xylans, a major component of plant cell walls, through specialized gene clusters that enhance their enzymatic activity [42]. The increased abundance of *Peptococcus* and *Prevotella* in BS birds aligns with existing evidence on microbiota dynamics in chickens. *Prevotella*, associated with fibre-rich diets and host metabolic modulation, has been reported to vary with breed and husbandry system, with potential effects on performance traits such as lipid metabolism [43]. The occurrence of *Peptococcus* may reflect broader trends observed in related taxa like *Peptostreptococcaceae*, whose abundance is shaped by age and environmental factors [44]. Studies on slow-growing genotypes, such as Sasso-T451A, have shown a more complex microbial community compared to commercial broilers, often including both *Prevotella* and *Peptococcus* [45]. These patterns are consistent with the established influence of rearing conditions and age on gut microbiota composition, particularly the enhanced microbial diversity observed in free-range systems [46]. The observed differences in the composition of the intestinal microbiota of chickens fit into a broader framework that recognizes the crucial role of these microbial communities in modulating metabolic, immune and behavioural responses, directly influencing animal health, growth and productivity [47, 48]. The variability observed among breeds also underlines the highly dynamic nature of the intestinal microbiome, suggesting that specific microbial configurations may contribute to determining distinctive physiological and phenotypic adaptations in different genetic lines [23, 49]. Independently of breed, significant correlations were observed between specific VFAs and the relative abundance of distinct bacterial taxa. Notably, citric acid levels were positively associated with *Fimimorpha* and *Anaerobutyricum*, suggesting that these genera may participate in or respond to pathways related to organic acid turnover. A positive correlation between formic acid and *Megamonas* was also detected, which aligns with previous findings highlighting the fermentative capacity of *Megamonas* in carbohydrate metabolism [50]. Moreover, isovaleric acid, which is a branched chain fatty acid commonly produced from protein fermentation, was associated with *Peptococcus*, a genus known for its proteolytic capabilities [51]. These findings collectively underscore the presence of breed-dependent variations in intestinal bacterial communities, potentially reflecting differences in metabolism, diet adaptation, or immune function among the groups.

The physiological consistency observed in morphometric and digestibility data is further reflected in the VFA results, which revealed a considerable degree of inter-individual variability among birds, yet no clear differences emerged among breeds. This suggests similar digestive patterns in line with the results of protein and fat digestibility and a comparable microbial fermentation profile across genotypes, pointing to a shared capacity to adapt to the same rearing environment. VFAs such as acetic, formic, citric, and isovaleric acids are key end-products of microbial metabolism, contributing to energy homeostasis, gut barrier integrity, and immune modulation [52].

## Conclusion

This research sheds light on the initial performance results of three slower growing chicken breeds (BP, BS, and MP) in a free-range system. Overall, these findings suggest that these birds, when raised in the same environment, tend to perform similarly. Their blood profiles and feather corticosterone levels were similar, indicating that they tend to have good health and wellbeing from a physiological standpoint. Gut health did not differ among breeds, and gut structure was likewise comparable. These findings may relate to differences in traits associated with intestinal structure and suggest possible breed-specific digestive strategies, which could be explored further in the context of nutrition. In terms of gut microbiota, while certain overarching trends were retained across breeds, the particularity of gut and host, diet, and environment means that there are still gaps that need to be closed in regard to these microbial communities. In a broader perspective, the overall lack of significant breed differences across breeds regarding intestinal structure, inflammation, digestibility, and VFA profiles indicates that the main gut functions are likely conserved across chicken breeds.

## Supplementary Information

Supplementary Material 1.

## Data Availability

The authors declare that data are not deposited in an official repository. The data that support the study findings are available upon request.

## Abbreviations

BP: Bionda Piemontese
BS: Bianca di Saluzzo
MP: Millefiori piemontese
SW: Slaughter weight
RTCC: Ready–to–cook carcass
CMF: Corticosterone metabolites in feathers
VH: Villus height
VW: Villus width
CD: Crypt depth
VH:CD: Villus height to crypt depth ratio
SEM: Standard error of the mean
GGT: Gamma–glutamyl transferase
AST: Aspartate aminotransferase
ALT: Alanine aminotransferase
CP: Crude protein
EE: Ether extract
ATTDC: Apparent total tract digestibility coefficient
TiO₂: Titanium dioxide
VFA: Volatile fatty acids
ASV: Amplicon sequence variant
PCA: Principal component analysis
HPLC: High performance liquid chromatography
ELISA: Enzyme–linked immunosorbent assay
