# Supplementary material for: Comparative evaluation of native slow-growing roosters: focus on gut integrity and physiological traits
Source: BMC Vet Res. 2025 Oct 17;21:616. doi: 10.1186/s12917-025-05084-1 (PMC12534952; doi:10.1186/s12917-025-05084-1)
Supplement: Supplementary file 1 — Supplementary Material 1. [file 12917_2025_5084_MOESM1_ESM.docx]

**Figure S1.** Photos of roosters of the three breeds: Bionda Piemontese, Bianca di Saluzzo and Millefiori Piemontese (in order of appearance.

**
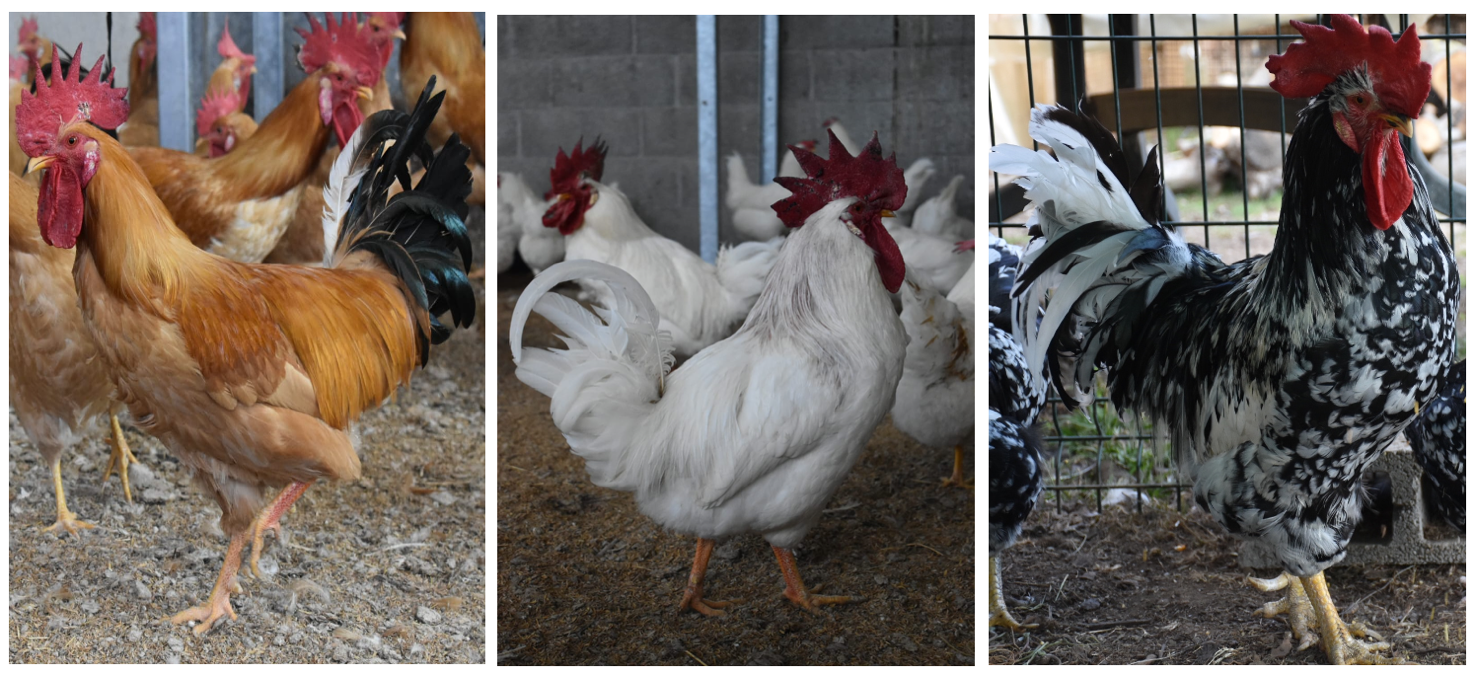
**

**Table S1.** Corticosterone metabolites in feathers (CMF) in roosters of Bionda Piemontese (BP), Bianca di Saluzzo (BS), and Millefiori Piemontese (MP) chicken breeds

|  | BP | BS | MF | SEM | P value |
| --- | --- | --- | --- | --- | --- |
| Corticosterone metabolites in feathers | 60.37 | 63.21 | 55.71 | 2.651 | 0.227 |

**Table S2.** Amplicon sequence variant (ASV) of bacteria in the gut of roosters of Bionda Piemontese (BP), Bianca di Saluzzo (BS), and Millefiori Piemontese (MP) chicken breeds

| Bacteria | BP | BS | MP | SEM | P value |
| --- | --- | --- | --- | --- | --- |
| *Anaerotignaceae* | 0.1885 | 0.2221 | 0.2487 | 0.01882 | 0.222 |
| *Atopobiaceae* | 0.5987 | 0.6192 | 0.3444 | 0.07228 | 0.018 |
| *Bacteroidaceae* | 0.5344 | 0.4277 | 0.6985 | 0.04493 | 0.026 |
| *Butyricicoccaceae* | 1.7544 | 1.9354 | 1.9108 | 0.28483 | 0.839 |
| *Coprobacillaceae* | 0.4195 | 0.5662 | 0.3867 | 0.03316 | 0.019 |
| *Eggerthellaceae* | 0.2718 | 0.3231 | 0.3433 | 0.02977 | 0.560 |
| *Elusimicrobiaceae* | 0.1885 | 0.1333 | 0.1287 | 0.06220 | 0.596 |
| *Gastranaerophilaceae* | 0.0474 | 0.0485 | 0.1672 | 0.01720 | 0.018 |
| *Lachnospiraceae* | 3.4095 | 3.6072 | 3.3864 | 0.24624 | 0.730 |
| *Muribaculaceae* | 0.6249 | 0.3549 | 0.1341 | 0.16258 | 0.010 |
| *Oscillospiraceae* | 0.9838 | 0.9354 | 1.2551 | 0.10423 | 0.202 |
| *Peptostreptococcaceae* | 1.0790 | 0.8790 | 0.6210 | 0.20598 | 0.228 |
| *Ruminococcaceae* | 0.8500 | 0.8513 | 0.7318 | 0.05535 | 0.471 |
| *Acutalibacter* | 0.3182 | 0.3397 | 0.3767 | 0.03797 | 0.485 |
| *Agathobaculum* | 0.6885 | 0.9490 | 0.7792 | 0.08190 | 0.362 |
| *Akkermansia* | 0.0203 | 0.0123 | 0.1700 | 0.01862 | 0.103 |
| *Alistipes* | 3.6782 | 4.6879 | 4.4921 | 0.30178 | 0.344 |
| *Anaerobutyricum* | 0.5713 | 0.6831 | 0.6700 | 0.05662 | 0.202 |
| *Anaerostipes* | 0.1805 | 0.2154 | 0.1669 | 0.03348 | 0.606 |
| *Aphodousia* | 0.6495 | 0.7444 | 0.8928 | 0.08394 | 0.270 |
| *Bacteroides* | 2.2803 | 2.1213 | 2.2287 | 0.18050 | 0.900 |
| *Barnesiella* | 0.9944 | 1.1095 | 1.2564 | 0.12736 | 0.358 |
| *Bifidobacterium* | 0.1223 | 0.2610 | 0.0938 | 0.03359 | 0.100 |
| *Blautia* | 1.9528 | 1.9308 | 1.6162 | 0.15766 | 0.217 |
| *Borkfalkia* | 0.4328 | 0.5785 | 0.7844 | 0.08275 | 0.011 |
| *Butyricicoccus* | 0.2531 | 0.2546 | 0.2274 | 0.06449 | 0.940 |
| *Cloacibacillus* | 0.9744 | 0.8738 | 1.3538 | 0.20747 | 0.136 |
| *Clostridium* | 0.5638 | 0.5477 | 0.6923 | 0.08089 | 0.377 |
| *Collinsella* | 0.4287 | 0.6685 | 0.4959 | 0.05500 | 0.224 |
| *Coprobacter* | 0.8772 | 0.7162 | 0.7946 | 0.12351 | 0.475 |
| *Desulfovibrio* | 1.0072 | 1.1864 | 1.2841 | 0.16321 | 0.448 |
| *Enterococcus* | 0.5928 | 0.7285 | 0.8246 | 0.11799 | 0.833 |
| *Erysipelatoclostridium* | 0.3869 | 0.6490 | 0.4367 | 0.04113 | 0.123 |
| *Eubacterium* | 0.5754 | 0.4341 | 0.4892 | 0.06027 | 0.361 |
| *Evtepia* | 0.3167 | 0.2895 | 0.3413 | 0.02969 | 0.516 |
| *Faecalibacterium* | 4.4269 | 4.0592 | 3.8315 | 0.43431 | 0.619 |
| *Faecalicoccus* | 0.3267 | 0.1749 | 0.2697 | 0.08522 | 0.402 |
| *Faecousia* | 2.1949 | 2.2197 | 2.4636 | 0.29448 | 0.587 |
| *Fimenecus* | 0.0977 | 0.0956 | 0.0613 | 0.06152 | 0.775 |
| *Fimimorpha* | 1.0567 | 1.5462 | 1.0315 | 0.19319 | 0.135 |
| *Frisingicoccus* | 0.0244 | 0.0149 | 0.0862 | 0.00493 | 0.209 |
| *Fusobacterium* | 0.1149 | 0.0910 | 0.1410 | 0.03788 | 0.795 |
| *Gemmiger* | 1.1026 | 1.3864 | 1.0400 | 0.16308 | 0.211 |
| *Helicobacter* | 0.1705 | 0.0849 | 0.0449 | 0.05905 | 0.105 |
| *Hydrogeniiclostridium* | 0.1277 | 0.1800 | 0.1736 | 0.01979 | 0.338 |
| *Lactobacillus* | 3.9536 | 1.8641 | 4.7446 | 0.99281 | 0.051 |
| *Lawsonibacter* | 0.8918 | 0.8297 | 0.7913 | 0.07238 | 0.504 |
| *Ligilactobacillus* | 2.7367 | 2.1141 | 2.9941 | 1.01890 | 0.690 |
| *Limiplasma* | 0.1018 | 0.1228 | 0.1941 | 0.02162 | 0.310 |
| *Limosilactobacillus* | 1.4974 | 1.0736 | 1.9769 | 0.39734 | 0.489 |
| *Mediterraneibacter* | 11.4344 | 11.7113 | 8.9336 | 1.02690 | 0.032 |
| *Megamonas* | 1.1756 | 0.6131 | 0.1754 | 0.44191 | 0.073 |
| *Merdibacter* | 0.5349 | 0.4197 | 0.3644 | 0.07399 | 0.113 |
| *Negativibacillus* | 0.4100 | 0.4813 | 0.3969 | 0.03547 | 0.168 |
| *Oliverpabstia* | 0.1503 | 0.1128 | 0.1536 | 0.03696 | 0.591 |
| *Onthenecus* | 0.4605 | 0.4649 | 0.6064 | 0.05979 | 0.505 |
| *Paludicola* | 0.6038 | 0.6195 | 0.6790 | 0.04128 | 0.522 |
| *Parabacteroides* | 1.7533 | 1.2436 | 1.3226 | 0.16073 | 0.149 |
| *Peptococcus* | 0.7905 | 1.1415 | 0.5649 | 0.08109 | 0.004 |
| *Phascolarctobacterium* | 2.2746 | 2.7479 | 1.8251 | 0.21712 | 0.009 |
| *Phocaeicola* | 18.6118 | 17.4739 | 19.6744 | 1.28096 | 0.381 |
| *Prevotella* | 1.3954 | 1.7128 | 1.0123 | 0.20206 | 0.033 |
| *Rikenella* | 0.1395 | 0.1233 | 0.2513 | 0.03136 | 0.039 |
| *Streptococcus* | 0.2787 | 1.0764 | 0.1787 | 0.15868 | 0.065 |
| *Turicibacter* | 0.4605 | 0.5392 | 0.3549 | 0.08021 | 0.568 |
